# A Yeast Modular Cloning (MoClo) Toolkit Expansion for Optimization of Heterologous Protein Secretion and Surface Display in *Saccharomyces cerevisiae*

**DOI:** 10.1021/acssynbio.3c00743

**Published:** 2024-03-14

**Authors:** Nicola M. O’Riordan, Vanja Jurić, Sarah K. O’Neill, Aoife P. Roche, Paul W. Young

**Affiliations:** † School of Biochemistry and Cell Biology, University College Cork, Cork T12 YN60, Ireland; ‡ AMBER Centre, Environmental Research Institute, University College Cork, Cork T23 XE10, Ireland

**Keywords:** Saccharomyces cerevisiae, modular cloning, MoClo toolkit, protein secretion, yeast surface display, sweet proteins

## Abstract

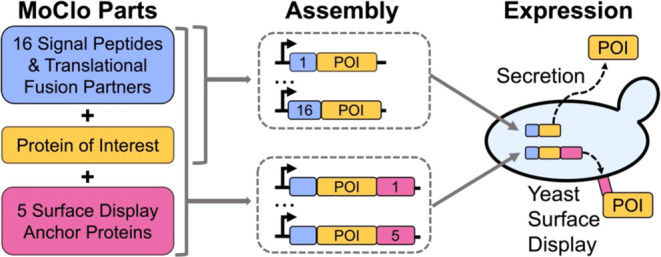

*Saccharomyces cerevisiae* is an attractive host for the expression of secreted proteins in a biotechnology context. Unfortunately, many heterologous proteins fail to enter, or efficiently progress through, the secretory pathway, resulting in poor yields. Similarly, yeast surface display has become a widely used technique in protein engineering but achieving sufficient levels of surface expression of recombinant proteins is often challenging. Signal peptides (SPs) and translational fusion partners (TFPs) can be used to direct heterologous proteins through the yeast secretory pathway, however, selection of the optimal secretion promoting sequence is largely a process of trial and error. The yeast modular cloning (MoClo) toolkit utilizes type IIS restriction enzymes to facilitate an efficient assembly of expression vectors from standardized parts. We have expanded this toolkit to enable the efficient incorporation of a panel of 16 well-characterized SPs and TFPs and five surface display anchor proteins into *S. cerevisiae* expression cassettes. The secretion promoting signals are validated by using five different proteins of interest. Comparison of intracellular and secreted protein levels reveals the optimal secretion promoting sequence for each individual protein. Large, protein of interest-specific variations in secretion efficiency are observed. SP sequences are also used with the five surface display anchors, and the combination of SP and anchor protein proves critical for efficient surface display. These observations highlight the value of the described panel of MoClo compatible parts to allow facile screening of SPs and TFPs and anchor proteins for optimal secretion and/or surface display of a given protein of interest in *S. cerevisiae*.

## Introduction

*Saccharomyces cerevisiae* has been used extensively as a host organism for heterologous protein expression due to factors, such as amenability to large-scale fermentation, capacity for eukaryotic post-translational modifications, and well-characterized genetics. Its “generally recognized as safe” (GRAS) status is especially important for proteins with applications in the food and biopharmaceutical sectors. Lower costs and decreased risk of viral contamination are potential advantages of *S. cerevisiae* over mammalian cells for therapeutic protein and antibody production, for example.^1,2^ In addition, *S. cerevisiae*-based yeast display technology has found many research and biotechnological applications for protein engineering.^3^ Nevertheless, many proteins are difficult to express at high levels in *S. cerevisiae* and achieving efficient secretion and/or cell surface display of heterologous proteins is often challenging.^3,4^

Protein secretion in *S. cerevisiae* is usually initiated by an amino-terminal signal peptide (SP) that is ultimately cleaved from the mature protein. SPs direct secreted proteins into the endoplasmic reticulum (ER) via either post-translational or cotranslational translocation mechanisms (e.g., the Sec- and SRP-dependent pathways, respectively). The pre-region of an SP precedes the signal peptidase cleavage site and has a tripartite structure generally consisting of a positively charged N-region, a hydrophobic α-helical H-region, and the C-region containing the cleavage site (often after an Ala residue).^5^ For many proteins, a pro-region, after the signal peptidase cleavage site, is also important for efficient secretion. The pro-region is typically removed from the mature protein by additional proteases, such as the Golgi-resident KEX2 protease, for example.^5,6^

Significant efforts have been made to improve the secretion of recombinant proteins by *S. cerevisiae*, including various strategies to identify sequences that would direct efficient secretion of a wide range of recombinant proteins. Mori et al.^7^ screened a library of 60 *S. cerevisiae* SPs and identified six that promoted high levels of secretion of an exogenous β-galactosidase enzyme. Holec et al.^8^ conducted a proteome-scale screen to identify SPs that gave a consistent, high-level surface display of proteins with diverse amino-terminal sequences. Other studies have optimized the widely used α-factor mating pheromone pre-pro-SP through a variety of strategies to achieve enhanced secretion of diverse proteins of interest.^9−12^ Bae and co-workers^13^ conducted a genome-wide screen to identify sequences termed translational fusion partners (TFPs) that enhanced the expression and/or secretion of several difficult-to-produce proteins. The TFPs identified typically contain additional amino acid sequences beyond the amino-terminal SP that may promote the correct folding of the recombinant protein to which they are fused or otherwise promote its progression through the secretory pathway. A variety of strategies have also been employed to improve the cell surface display of proteins of interest. These include signal peptide optimization and varying the choice of display anchor proteins used (reviewed in ref (3)). Overall, while the aforementioned studies identify many promising approaches that can greatly enhance secretion or cell surface display in *S. cerevisiae*, no one strategy is likely to be optimal for every heterologous protein of interest.

In the absence of a universal secretion promoting sequence that will work for all recombinant proteins, an efficient system to rapidly sample a library of such sequences is highly desirable. The same principle applies to the selection of anchor proteins for yeast display. Modular cloning (MoClo) is a standardized, hierarchical system that facilitates the efficient assembly of plasmids containing single or multiple transcriptional units using type IIS restriction enzymes.^14^ The MoClo strategy has been widely adopted^15^ and has also been adapted for specific applications, including for use in *S. cerevisiae* with the development of a modular cloning yeast toolkit (MoClo-YTK).^16^ Here, we have formatted a variety of secretion enhancing and surface display sequences described in the scientific literature for use in conjunction with the yeast MoClo toolkit in a way that permits the production of recombinant proteins with native (or near-native) amino-terminal sequences following SP or TFP cleavage. The resulting collection of plasmids constitutes a yeast secretion/display (YSD) toolkit that is being made available to the research community.

## Results and Discussion

### Expansion of the MoClo-YTK to Accommodate a Panel of Secretion Promoting Sequences

**Figure 1:**
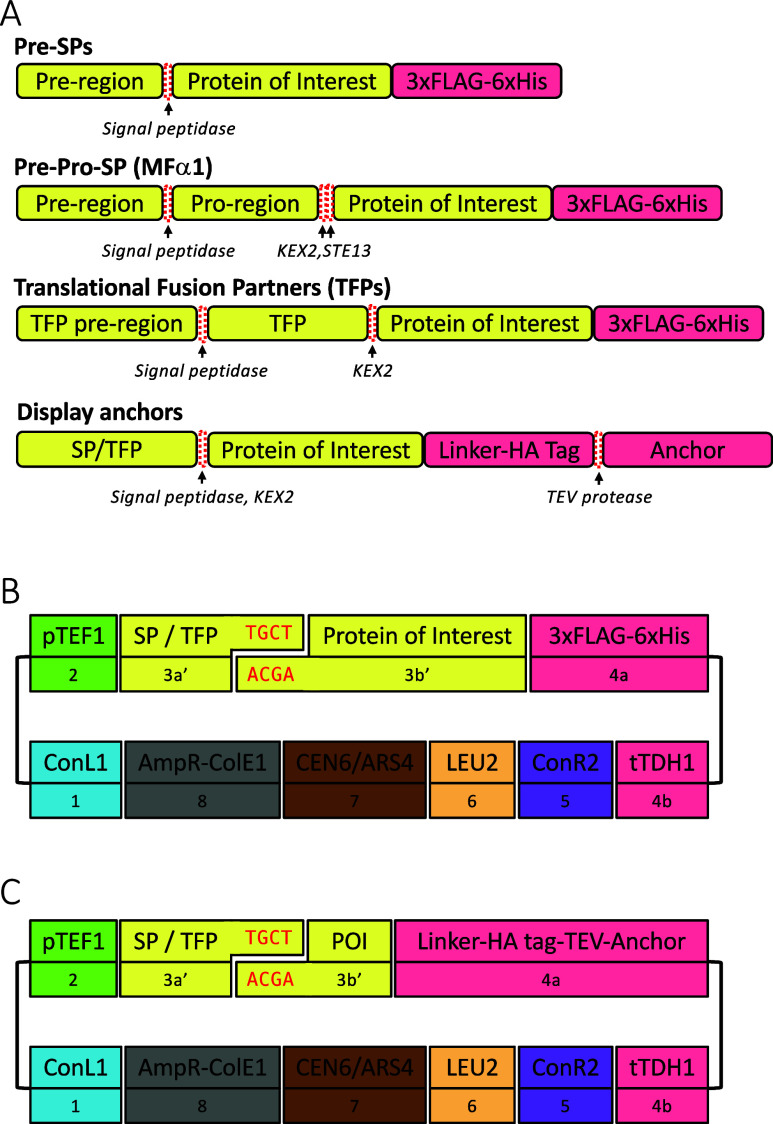
Schematic representation of constructs and assembly strategy. (A) The indicated types of amino-terminal secretion promoting sequences were combined with a protein of interest and a carboxyl-terminal 3xFLAG-6xHistidine tag to assess their secretion efficiency or an HA epitope-tagged anchor protein for surface display experiments. (B) Yeast expression constructs to evaluate a panel of SPs and TFPs were assembled using the standard parts from the yeast modular cloning toolkit, with the exception of the parts denoted as 3a′ and 3b′ that are joined by a custom overhanging sequence (red text). (C) Similar expression constructs were used to evaluate yeast surface display anchors, which were assembled as standard 4a part types and encoded a flexible linker (GGGGSGGGGS), an HA epitope tag, and a TEV protease cleavage site upstream of the anchor protein. POI = protein of interest.

The MoClo yeast toolkit includes a selection of promoter and terminator elements that can be combined with a coding sequence to generate transcriptional units for protein expression in vectors with several possible selectable markers and origins of replication. We sought to expand this toolkit by developing SP and TFP sequences as “parts” that can be readily combined with a coding sequence of interest to optimize protein secretion or surface display (Figure 1A). Maintaining the native amino- and/or carboxyl-terminal amino acid sequence can be critical to the functionality of recombinantly expressed proteins and may also be important for regulatory reasons. With this in mind, we considered the sequence of yeast SP pre-sequences, which most frequently have an alanine residue at the −1 position relative to the signal peptidase cleavage site as part of an Ala-X-Ala or Val-X-Ala motif.^17^ To join SP pre-sequences ending with Ala to coding sequences, without introducing any extra amino acids, we chose a TGCT overhang for DNA assembly, in which the GCT codes for Ala (Figure 1B). A TGCT overhang is predicted to have high fidelity when used as part of the YTK toolkit when analyzed using the NEBridge GetSet tool (Figure S1). This approach ensures that coding sequences with their native amino termini will be generated following signal peptidase cleavage of the SP pre-sequence.

We initially selected a panel of nine SP pre-sequences, three TFPs, and an engineered mating factor α pre-pro-sequence (MFαpp8) that was previously reported to enhance antibody secretion^11^ (Table 1). The selected sequences were designed as yeast modular cloning parts that are equivalent to subtype 3a parts according to the convention of Lee et al.^16^ except using a TGCT rather than TTCT overhang at the 3*a*/3b junction (Figure 1B). We denote these parts as 3a′ and the coding sequence parts as 3b′ to highlight this distinction. The choice of TGCT overhang necessitated changing glycine to alanine at the −1 position for two SP pre-sequences and an amino acid change at the −2 position for one of these (Table 1^18^). For the MFαpp8 sequence, a Glu-Ala to Asp-Ala change was made for similar reasons at the second STE13 protease cleavage site. A recognition sequence for KEX2 protease (Leu-Asp-Lys-Arg) was included after the TFP sequences such that a recombinant protein with just an additional amino-terminal alanine is expected to be generated following proteolytic cleavage.

**Table 1:**
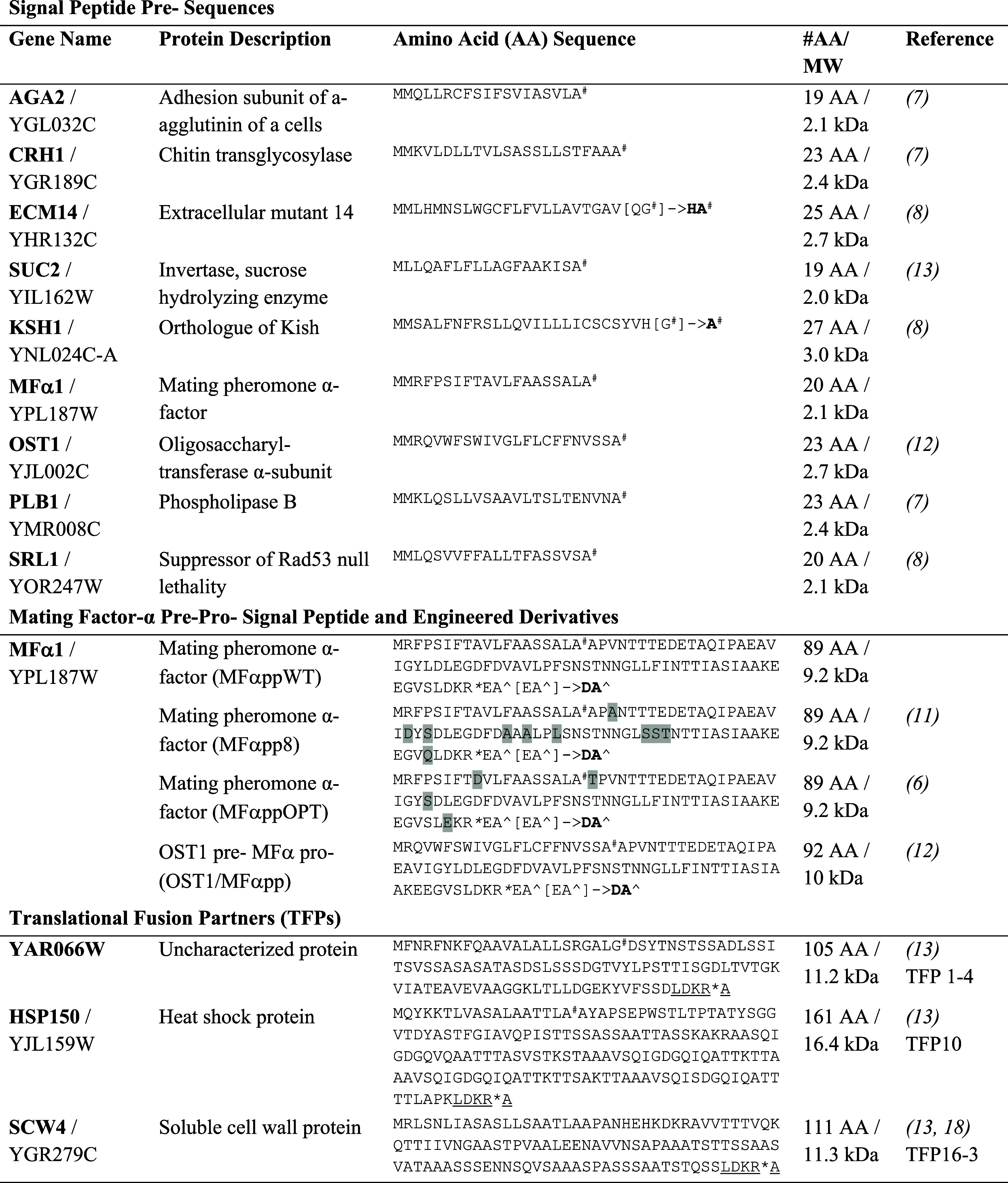
Signal Peptides and Translational Fusion Partners Used in This Study^a^

a The expected positions of cleavage by signal peptidase (#), STE13 protease (∧), and KEX2 protease (*) are indicated. The KEX2 protease recognition sequence and an additional alanine residue added to the TFP sequences are underlined. Instances in which the amino acid sequence of the signal peptide was changed as a result of the DNA overhang used for modular cloning are indicated with the original sequence in square brackets and the modified sequence in bold. Signal peptidase cleavage sites that could be predicted confidently for two TFPs using SignalP 6.0^19^ are indicated. Mutated residues in MFαpp8 and MFαppOPT are highlighted.

### Evaluation of the Panel of Secretion Promoting Sequences

Expression vectors were assembled for each SP and TFP using a strong constitutive pTEF1 promoter (Figure 1B). A type 4a part encoding a carboxyl-terminal 3xFLAG-6xHistidine tag was included to facilitate the detection or purification of the recombinant protein. As an initial test of the system, the expression of a known *S. cerevisiae* secreted protein, invertase (SUC2), was evaluated by Western blotting of culture media (Figure S2). Secreted invertase was detected for all SP and TFP sequences, indicating that they were all functional when used in conjunction with a readily secreted endogenous yeast protein.

**Figure 2:**
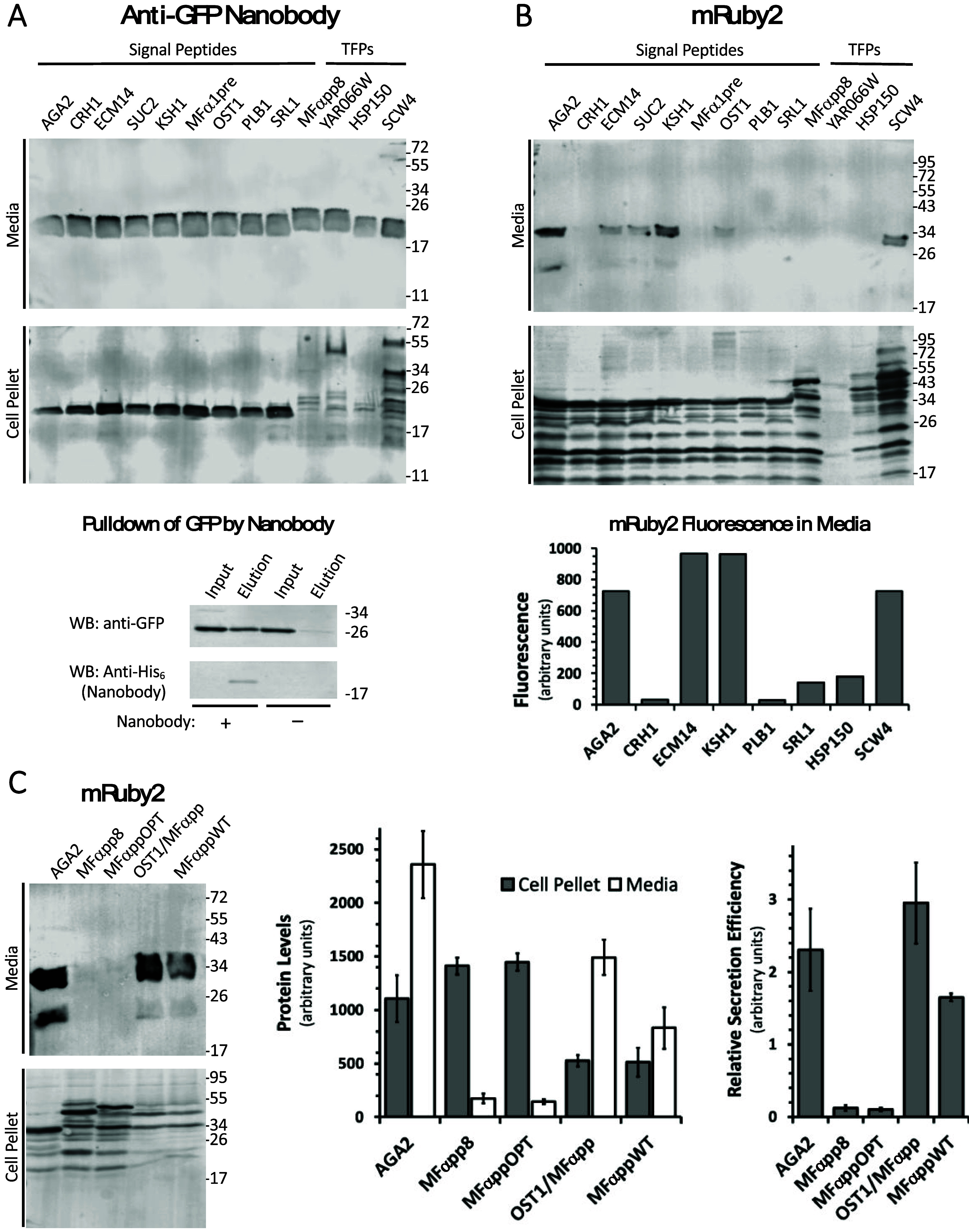
Evaluation of a panel of secretion promoting sequences for the expression of a nanobody and the mRuby2 fluorescent protein. Recombinant proteins were targeted for secretion using the indicated SPs and TFPs. Secreted and intracellular proteins were detected from the media and cell pellet, respectively, by Western blotting for a carboxyl-terminal 6xHis tag. (A) An anti-GFP nanobody was readily secreted using all SPs and TFPs (upper panel) and was functional, as assessed by its ability to pull-down GFP from a cell lysate of GFP-expressing HEK293T cells (lower panel). (B) Only half of the SPs and one TFP directed efficient secretion of the mRuby2 fluorescent protein, as assessed by Western blotting (upper panel). Fluorescence measurements of cell-free media confirmed this pattern for a subset of the SPs and TFPs (lower panel). (C) Wildtype and engineered variants of the MFα pre-pro-SP differ significantly in their ability to direct secretion of mRuby2 as assessed by Western blotting (left panel). Protein levels from three independent experiments were quantified (middle panel) and the ratio of levels observed in media versus cell pellet samples was calculated as a measure of relative secretion efficiency (right panel). Sizes of molecular weight markers are indicated in kDa. Error bars represent SEM.

We then tested the system for production of nanobodies, variable domains of heavy-chain-only antibodies from camelid species that have widespread applications as imaging reagents and therapeutics. An anti-green fluorescent protein (GFP) nanobody^20^ was readily secreted into the culture media using all of the SPs and TFPs (Figure 2A). Intracellularly, for all pre-SPs, the nanobody was detected as a relatively discrete band at the expected ∼18 kDa molecular weight of the pre-protein, while the MFαpp8 SP and three TFPs resulted in multiple forms of the intracellular nanobody resolving at higher molecular weights. When purified from the media, the nanobody was able to efficiently bind to GFP in a pull-down assay, verifying its functionality (Figure 2A, lower panel).

Next, we examined the secretion of the fluorescent protein mRuby2^21^ (Figure 2B). In contrast to the nanobody, efficient secretion of mRuby2 was only observed with the pre-SPs AGA2, ECM14, SUC2, KSH1, and OST1 and the TFP SCW4. Intracellularly, relatively uniform levels of mRuby2 were detected for all SPs and TFPs except YAR066W. Differences in mRuby2 levels in the media therefore seem to reflect the inability of some pre-SPs and TFPs to direct mRuby2 through the secretory pathway rather than a failure of protein expression. Secreted mRuby2 is functional, as indicated by fluorescence measurements of the media in separate experiments using a subset of the SPs and TFPs, with the same pattern of secretion efficiency being apparent (Figure 2B, lower panel).

Neither the MFα1 pre- nor the engineered MFα1 pre-pro-sequence MFαpp8 directed efficient secretion of mRuby2. Several other improved MFα1 pre-pro-sequences have been described in the literature. We were curious to examine the performance of two of these in promoting secretion of mRuby2 in comparison to MFαpp8 and the wildtype MFα1 pre-pro-sequence (Table 1). MFαppOPT arose from a systematic attempt to design a universal signal peptide^6^ and has four amino acid substitutions compared to the wildtype sequence. By contrast, OST1-MFαpp is a fusion of the OST1 pre-sequence with the MFα1 pro-sequence that was used to promote secretion of a monomeric superfolding GFP variant^12^ and three distinct exogenous proteins in *S. cerevisiae*(9) via cotranslational targeting to the ER. Secretion of mRuby2 directed by the wildtype MFα pre-pro-SP, MFαppOPT, MFαpp8, and OST1/MFαpp was compared to the AGA2 pre-SP—the most efficient sequence from our initial panel. OST1/MFαpp and MFαppWT directed reasonable levels of mRuby2 secretion into the media, though not as high as those for AGA2. Only minimal secretion was observed for MFαpp8 and MFαppOPT. Protein levels in the cell pellet were lower for OST1/MFαpp and MFαppWT compared with the other sequences. Calculating the ratio of mRuby2 levels in the media versus the cell pellet as a measure of relative secretion efficiency revealed OST1/MFαpp to be on par with AGA2, while MFαppWT was slightly less efficient but still dramatically better than either of the engineered MFαpp8 or MFαppOPT sequences.

The profiles of secretion observed for the anti-GFP nanobody and mRuby2 differ significantly. The former was efficiently secreted, regardless of the secretion promoting sequence employed. By contrast, only six of 13 sequences in our initial panel directed appreciable secretion of the mRuby2 fluorescent protein with the pre-SPs AGA2, and KSH1 and TFP, SCW4 giving the highest levels of secretion. Secretion by *S. cerevisiae* of mRuby2 has not, to our knowledge, been previously described in the literature. However, difficulties in achieving secretion of GFP from *S. cerevisiae* have been reported^22^ and it has been suggested that SPs, such as OST1, that direct the recombinant protein toward the cotranslational translocation pathway at the ER are preferable for secretion of some fluorescent proteins.^12^ While the OST1 pre-SP directed low levels of secretion of mRuby2 in this study, the hybrid OST1/MFαpp was more successful. Surprisingly, the engineered MFαpp8 and MFαppOPT sequences, which have been shown to enhance the secretion of numerous proteins, failed to direct efficient secretion of mRuby2, while the wildtype sequence from which they were derived was relatively successful. MFαpp8, in particular, has been routinely used in place of the wildtype sequence, but our observations caution against the assumption that engineered SPs are always an improvement over wildtype sequences for every protein of interest.

### Application of the Toolkit Expansion for Secretion of Sweet Proteins

**Figure 3:**
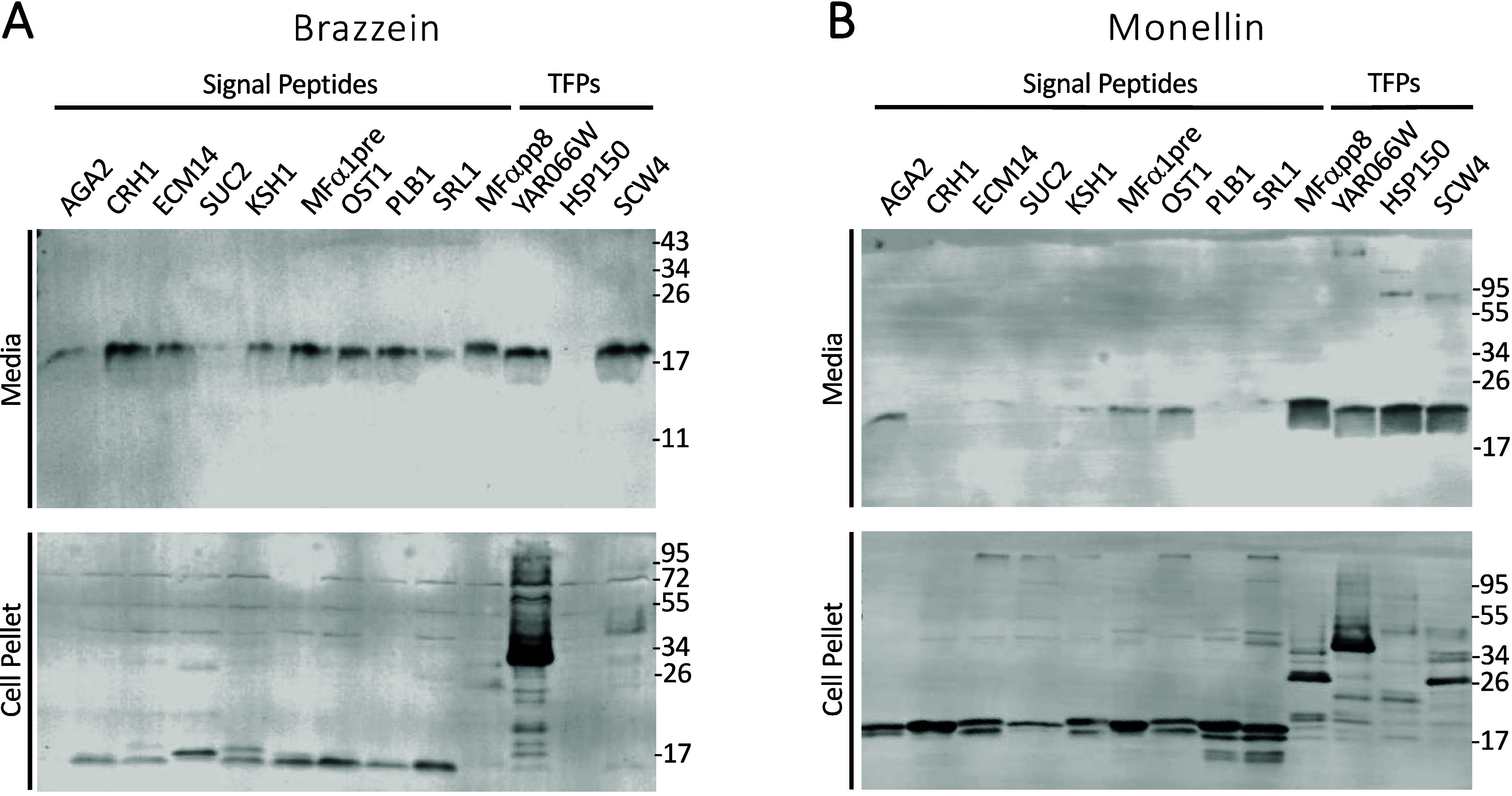
Application of the SP/TFP panel for the recombinant production of sweet proteins. Expression and secretion of recombinant sweet proteins were assessed by Western blotting as before. (A) Most, but not all, SPs and TFPs promoted moderate levels of brazzein secretion. (B) Marked differences in the SPs and TFPs that gave optimal secretion of single-chain monellin were apparent. Sizes of molecular weight markers are indicated in kDa.

Sweet proteins are potential low-calorie alternatives to sugar and artificial sweeteners, and *S. cerevisiae* is an attractive host for their production given its widespread use in the food and beverage industry.^23^ We therefore applied the panel of pre-SPs and TFPs for the secretion of two sweet proteins brazzein^24^ and monellin.^25^ Brazzein was expressed at rather low levels overall; nonetheless, all SPs and TFPs from the panel except for HSP150 promoted brazzein secretion (Figure 3A). The secreted protein was observed as a broad band running at, or just above, the 17 kDa molecular weight marker higher—somewhat higher than its predicted ∼10 kDa molecular weight. Little to no intracellular protein was detected for HSP150, explaining the absence of secreted brazzein for this TFP. Interestingly, high levels of intracellular protein for YAR066W compared to the SCW4 TFP or the pre-SPs, for example, did not result in greater brazzein secretion.

The second sweet protein tested was an engineered single-chain version of monellin (sc-monellin) termed MNEI.^25,26^ Secreted sc-monellin was detected as a broad band, slightly higher than its ∼17 kDa predicted molecular weight when expressed with pre-SPs AGA2, ECM14, KSH1, MFα1, OST1, and SRL1. Meanwhile, little to no secretion was observed with CRH1, SUC2, and PLB1, despite similar amounts of intracellular protein. The best levels of sc-monellin secretion were observed when it was expressed with the MFαpp8 pre-pro-SP and the TFPs; however, higher molecular weight bands were observed in the media for the TFPs. Intracellularly, variable levels of sc-monellin were detected for all SPs and TFPs with little apparent correlation between these levels and the amount of secreted protein detected in the media.

Comparing the secretion profiles of brazzein and sc-monellin, we observed variable degrees of brazzein secretion for 12 of the 13 sequences in our initial panel, while minimal if any expression or secretion was seen for the TFP HSP150. For sc-monellin, on the other hand, the three TFP sequences outperformed the pre-SPs with HSP150 and MFαpp8, exhibiting the best levels of protein secretion overall. The contrasting performance of the HSP150 TFP for production of these two sweet proteins underscores the difficulty of extrapolating the efficiency of a secretion promoting sequence from one protein of interest to another.

Glycosylation of heterologous proteins targeted for secretion in *S. cerevisiae* has been reported and has the potential to affect protein function. We note that the secreted nanobody, brazzein, and sc-monellin proteins were observed as broad bands by Western blot that migrated slightly higher than the expected molecular weight, while secreted mRuby2 was observed as a doublet band. These variations in the molecular weight might be explained by variable degrees of glycosylation. Very few glycosylation sites are predicted for these proteins using *NetNGlyc1.0*(27) and *NetOGlyc4.0*(28) (Table S1). However, these tools are primarily intended for use with mammalian proteins and prediction of *S. cerevisiae* O-mannosylation sites in particular may not be reliable.^29,30^ Variable O-mannosylation on serine or threonine residues not predicted by *NetOGlyc4.0* is therefore plausible. Glycosylation would appear to explain the high-molecular-weight proteins detected intracellularly for the MFαpp8 SP and TFPs since predictions and experimental data from the literature support N-glycosylation of MFα pre-pro-sequence and O-mannosylation of the three TFP sequences.^27−31^ Extensive glycosylation of secreted invertase has also been described^32,33^ and agrees with our observations (Figure S2).

Sweet proteins have potential applications as low-calorie sweeteners. Obtaining brazzein or monellin from the native plants that produce them (*Pentadiplandra brazzeana* and *Dioscoreophyllum cumminsii*, respectively) has not proven to be commercially viable and so there is considerable interest in their heterologous production in either established crop plants or in microorganisms amenable to industrial fermentation.^34^ Single-chain monellin has greater thermal stability compared to the native protein consisting of two polypeptide chains. Sc-monellin was successfully secreted in *S. cerevisiae*, utilizing the MFα1 pre-pro-SP under the control of a GAL1 promoter.^35^ The maximum yield achieved was 0.41 g/L, although the functionality of the secreted protein was not tested. We observed efficient secretion of sc-monellin directed by the MFαpp8 SP, however, high secretion levels were also observed for all three TFPs. Secretion levels for the TFP HSP150 were equivalent to those for MFαpp8 while showing less intracellular sc-monellin, suggesting that HSP150 might traffic sc-monellin more efficiently through the secretory pathway. There may be merit therefore in evaluating the yields of sc-monellin that could be obtained using HSP150 or other TFPs in a large-scale fermentation system, particularly if the choice of a promoter is also optimized (something which would be greatly facilitated by the promoter parts provided in the MoClo-YTK). Further work would also be necessary to characterize if the resulting secreted protein is functional, especially because Liu et al. found that a single-chain monellin secreted using the MFα1 SP did not taste sweet likely due to glycosylation.^36^

Brazzein with an amino-terminal Asp residue has optimal sweetness properties.^37^ Importantly, the custom TGCT overhang used here to join SP encoding 3a′ parts to 3b′ parts encoding proteins of interest facilitates this maintenance of native amino-terminal sequences following SP cleavage. Heterologous expression of brazzein has previously been achieved in transgenic plants, bacteria, *Escherichia coli* and *Lactococcus lactis*, and the yeasts, *S. cerevisiae*, *Pichia pastoris*, and *Kluyveromyces lactis*.^38^ Brazzein was expressed intracellularly in *S. cerevisiae* and observed by Western blotting at a higher than expected molecular weight of ∼35 kDa, which the authors attribute to possible hyper-glycosylation.^39^ In our hands, brazzein secreted from *S. cerevisiae* is resolved as a broad band running at, or just above, 17 kDa. This is somewhat higher than the predicted ∼10 kDa, which might suggest a degree of glycosylation, though less than that in the aforementioned report. An assessment of the glycosylation status and functionality of the secreted brazzein would therefore be of interest. Notably, while good brazzein secretion was achieved using the AGA2 and MFapp8 SPs, little or no protein was detected intracellularly. This suggests efficient targeting and passage of brazzein through the secretory pathway with these SPs, highlighting the potential value of examining both intracellular and secreted protein levels when evaluating secretion promoting sequences. In *P. pastoris*, secretion of functional brazzein was achieved utilizing various SPs derived from different origins,^40^ while in *K. lactis*, the *S. cerevisiae* MFα pre-pro- and MFα pre-SPs were used successfully to secrete functional brazzein.^41,42^ An existing *P. pastoris* MoClo toolkit contains a small panel of hybrid SPs that are based on the fusion of various pre-SP sequences to an engineered version of the *S. cerevisiae* MFα pre-pro-sequence.^43^ These hybrid SPs generally did not outperform the wildtype MFα sequence, however. In addition, a MoClo-YTK expansion compatible with another species belonging to the *Kluyveromyces* genus (*K. marxianus*) has been described.^44^ The broader panel of SPs and TFPs described here are compatible with these MoClo toolkits and so could potentially be applied to further optimize the secretion of brazzein, or other proteins of interest, in these other biotechnologically significant yeast species.

### Adaptation of the Toolkit Expansion to Accommodate a Panel of Display Proteins

Aside from the production of heterologous proteins, *S. cerevisiae* is widely used for yeast surface display, which has found particular application in protein engineering. Significant effort is often required to achieve an efficient surface display for a given protein with the choice of anchor protein being one of the variables that may need to be optimized.^3^ The use of modular cloning to combine our panel of SPs and TFPs with various anchor proteins has the potential to streamline this optimization process. We therefore developed a panel of five anchor proteins as type 4a parts according to MoClo-YTK convention^16^ (Figure 1C and Table 2). The panel includes the endogenous *S. cerevisiae* proteins Aga2p, Cwp2p, Sed1p, and Tip1p^45^ as well as a synthetic anchor protein called 649 stalk.^46^ They were designed as 4a parts with a linker sequence, an HA epitope tag, and a TEV protease cleavage site upstream of the anchor protein.

**Table 2:** Yeast Surface Display Anchors Used in This Study

Protein Name	Protein Description	**#AA/MW**	Reference
Aga2p	adhesion subunit of α-agglutinin of a cells	70 AA/7.6 kDa	(45)
Cwp2p	cell wall protein	72 AA/6.9 kDa	(45)
Sed1p	suppression of exponential defect	320 AA/32.7 kDa	(45)
Tip1p	temperature shock-inducible protein	191 AA/18.9 kDa	(45)
649 Stalk	649 Stalk + GPI Anchor	688 AA/67.1 kDa	(46)

**Figure 4:**
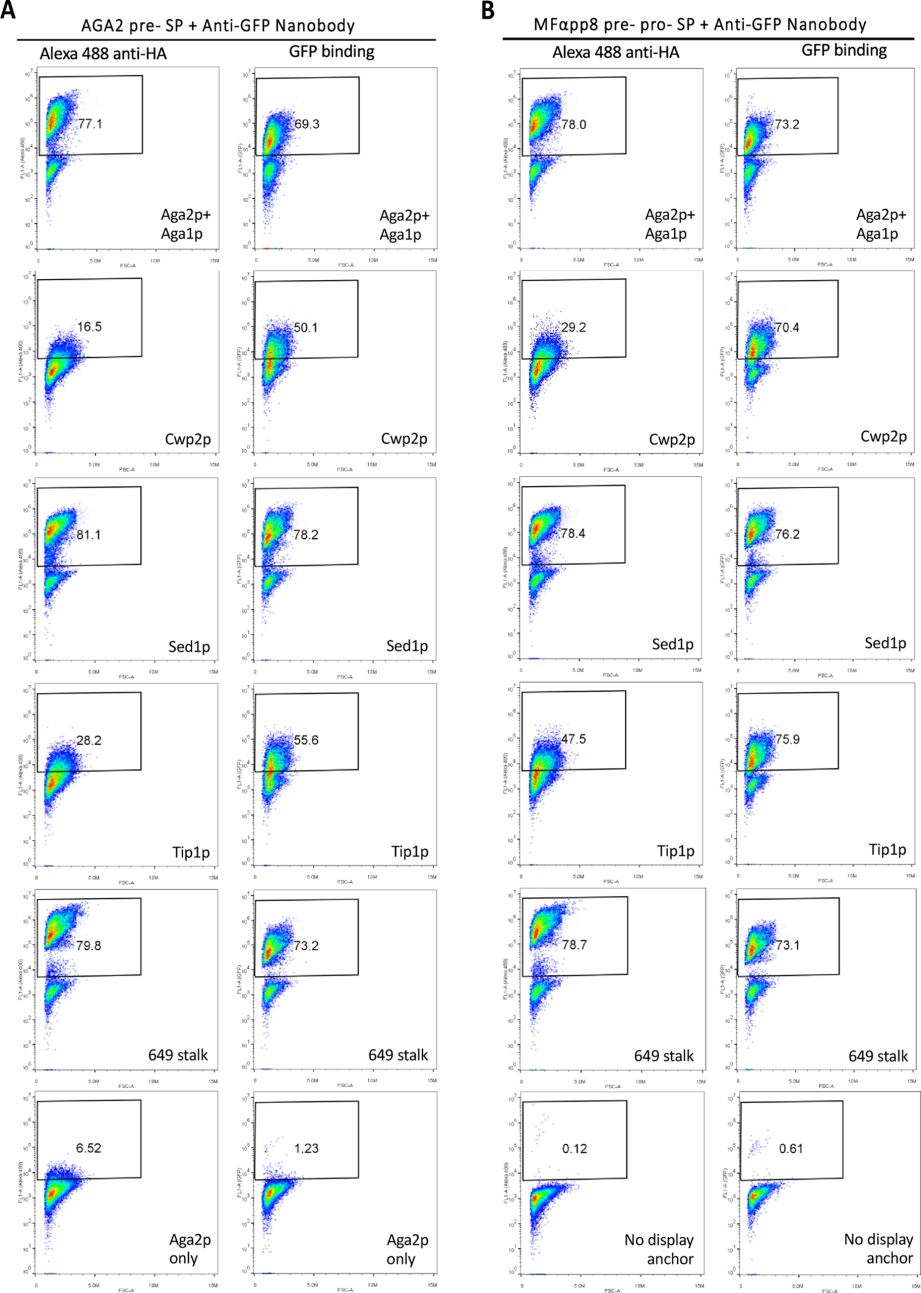
Evaluation of five anchor proteins for the surface display of an anti-GFP nanobody. The anti-GFP nanobody was expressed in yeast cells in combination with the indicated anchor proteins and either the AGA2 pre-SP (A) or the MFαpp8 pre-pro-SP (B). Surface display was assessed by flow cytometry using an Alexa-488 fluorophore-conjugated anti-HA antibody or the binding of GFP by the displayed nanobody. Untransformed BY4741 cells were used as negative control. Plots depict the FL1 signal (Alexa-488 or GFP fluorescence; *Y*-axis) versus forward light scattering (FSC; *X*-axis) with the percentage of positively stained cells indicated.

Expression constructs for the anti-GFP nanobody were assembled for each of the anchor proteins in combination with both the AGA2 pre- and MFαpp8 SPs. Surface display was analyzed by flow cytometry using an anti-HA-tag antibody (Figure 4). Aga2p had to be coexpressed with its binding partner Aga1p because of the low endogenous expression of Aga1p in BY4741 cells. The Aga2p, along with the Sed1p and 649 stalk anchors, exhibited a very efficient surface display of the nanobody for both SPs with >75% of cells positively stained. Cwp2p and Tip1p were somewhat less efficient overall but both showed notably better surface display when combined with MFαpp8 SP than AGA2 pre-SP. To confirm that the displayed nanobody was functional and that the linker connecting it to the anchor protein remained intact, we mixed yeast cells displaying the nanobody with cell lysate from *E. coli* cells expressing GFP. Flow cytometry analysis to detect cells with bound GFP showed very similar results to those obtained using the anti-HA-tag antibody for Aga2p, Sed1p, and the 649 stalk. Surprisingly, for cells expressing the nanobody coupled to the Cwp2p and Tip1p anchors, labeling with GFP was much more efficient than for the anti-HA-tag antibody. This may reflect greater accessibility of the smaller GFP molecules, allowing them to bind to the nanobody displayed by these shorter anchor proteins. Overall, the robust binding of GFP to cells expressing the different anchor proteins demonstrates the accessibility and functionality of the displayed nanobody.

**Figure 5:**
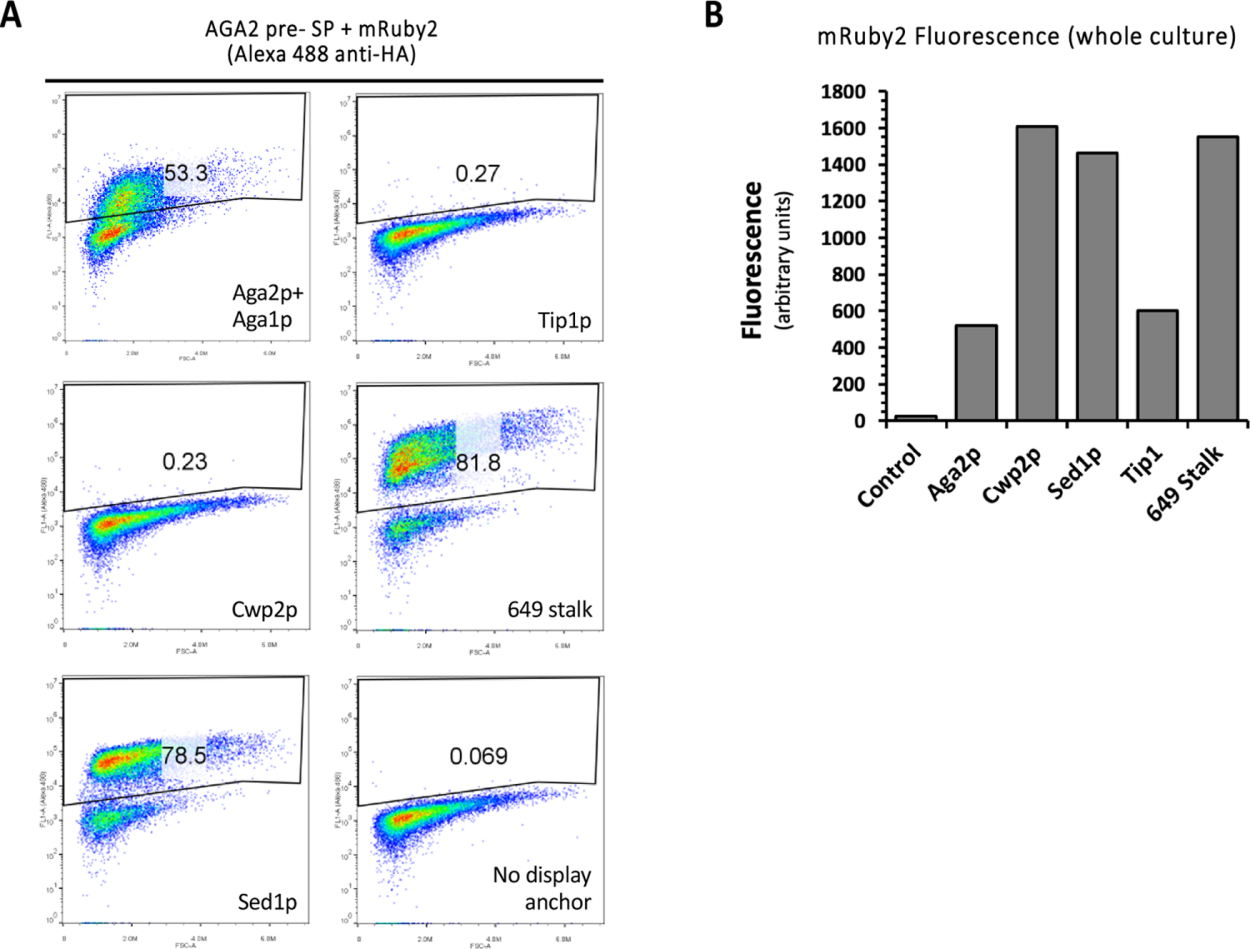
Evaluation of five anchor proteins for the surface display of mRuby2. mRuby2 was expressed in yeast cells in combination with the indicated anchor proteins and the AGA2 pre-SP. Surface display was assessed by flow cytometry using an Alexa-488 fluorophore-conjugated anti-HA antibody. Untransformed BY4741 cells were used as a negative control. Plots depict the FL1 signal (Alexa-488 or GFP fluorescence; *Y*-axis) versus forward light scattering (FSC; *X*-axis) with the percentage positively stained cells indicated (left panel). Measurements of mRuby2 fluorescence in whole cultures confirmed protein expression for all anchors (right panel).

We next examined the surface display of mRuby2 with each anchor protein using AGA2 pre-SP that had worked best for mRuby2 secretion. In this case, Sed1p and the 649 stalk gave efficient surface display of mRuby2, Aga2p was moderately effective, while virtually no surface display was seen with the Cwp2p and Tip1p anchors (Figure 5A). Fluorescence measurements of whole yeast cultures revealed strong mRuby fluorescence in all cases, indicating that the absence of surface display was not due to the lack of protein expression (Figure 5B).

Yeast surface display has long been used for protein engineering to optimize binding affinity, stability, and enzymatic activity.^47^ It has also been applied to the identification and study of protein–protein interactions, as well as the development of biosensors and whole-cell biocatalysts.^3^ For some applications, co-display of two or more proteins is required, for example, when expressing multi-enzyme complexes or for co-display of an enzyme along with a protein that increases association with a substrate.^48^ Achieving efficient display of proteins of interest as well as controlling their expression levels and localization within the cell wall can be critical to such experiments. The panels of anchor proteins and secretion promoting sequences described here, when combined with promoters and multigene assembly capacities of the yeast modular cloning toolkit and its expansions,^16,49^ should accelerate the design and execution of these more sophisticated applications of yeast surface display technology.

### Summary

The MoClo-YTK is a useful genetic engineering platform that permits a rapid modular multipart assembly of designated DNA part types. It was originally designed to permit facile genome editing, integration, and CRISPR applications^16^ and has since been expanded to achieve a wide range of further functionalities. Expansions include further CRISPR and genomic integration capacities, the capability to make combinatorial libraries,^50^ greater multiplexing capacity with more selectable markers, genomic integration sites and inducible promoters,^49^ and specific subcellular targeting of proteins.^51^ The YSD toolkit presented here constitutes another expansion of the MoClo-YTK, which enables the rapid evaluation of panels of SPs and TFPs and/or anchor proteins in order to optimize the secretion and/or surface display of a protein of interest. The secretion promoting sequences and anchor proteins have been curated from the most promising candidates in the literature on these topics. We suggest, however, that no one universal sequence or anchor protein is likely to be suitable for every application. The prior literature, as well as the examples presented here, indicates that for many proteins of interest, the optimal combination of secretion promoting sequences and/or anchor proteins must be determined empirically by experimentation and cannot be routinely predicted. For example, only three sequences from our initial panel, the SPs AGA2, OST1, and the TFP SCW4, performed well in directing the secretion of all four heterologous proteins of interest that we tested. However, sequences other than these three directed the highest secretion levels of the nanobody and sc-monellin. The four heterologous proteins of interest examined have diverse structural and biochemical characteristics but are all relatively small. We expect potentially greater variability of secretion efficiency for larger, multidomain proteins. Similarly for surface display, while the Aga2p, Sed1p, and 649 stalk anchors performed consistently well, the efficiency of surface display observed for the Cwp2p and Tip1p anchors was highly dependent on the SP used and the protein of interest being displayed. Overall, these observations underline the utility of screening a range of secretion promoting sequences or anchors for a given protein of interest. The YSD toolkit expansion seeks to harness efficiencies of the modular cloning framework in order to standardize this trial and error process and accelerate the design, build, and test cycle for synthetic biology experiments involving protein secretion or surface display in yeast.

## Methods

### Strains and Growth Conditions

The *S. cerevisiae* strain used was BY4741 (MATa his3Δ1 leu2Δ0 met15Δ0 ura3Δ0). For selection, cells were grown at 30 °C in minimal synthetic media or agar plates containing 6.8 g/L yeast nitrogen base without amino acids (Sigma-Aldrich), 2 g/L dextrose (Formedium), and a complete supplement mixture of amino acids minus leucine or minus leucine and histidine (MP Biomedicals). *E. coli* DH5α cells were used for all cloning steps and were grown at 37 °C on Lysogeny Broth (LB) agar plates or in liquid LB media with shaking at 200 rpm. 35 μg/mL chloramphenicol or 50 μg/mL carbenicillin was added as appropriate.

### Chemicals, Molecular Biology Reagents, and DNA Part Generation

All chemicals were obtained from Merck/Sigma-Aldrich, unless stated otherwise. The MoClo-YTK plasmid kit was a gift from John Dueber (Addgene kit # 1000000061). Enzymes used were from New England Biolabs, unless stated otherwise. Single-stranded oligonucleotides and double-stranded DNAs (gBlocks) were ordered from Integrated DNA Technologies. SP, TFP, display protein, and recombinant protein encoding parts were designed to be compatible with the MoClo Yeast Toolkit (YTK),^16^ with the exception of the overhang between 3a′ and 3b′ parts. Sequences of all parts are summarized in Supporting Information File S1. Smaller parts were generated by annealing and extending complementary oligonucleotides using Klenow polymerase. Briefly, 1 μL of a 100 μM stock of each oligonucleotide was mixed with 48 μL of water, heated at 95 °C for 5 min, and then cooled by 1.5 °C/min for 1 h. 16 μL of annealed oligonucleotide was incubated with 5 units of Klenow polymerase, 1 μL of dNTPs (Thermo Fisher Scientific), and 2 μL of T4 DNA Ligase Buffer at 25 °C for 15 min and then heat-inactivated at 75 °C for 20 min. For larger parts, PCR amplification from yeast genomic DNA, plasmids, or synthetic DNA fragments was performed using Phusion High Fidelity Polymerase (Thermo Fisher Scientific) according to the manufacturer’s protocol. PCR purifications, gel extractions, and plasmid minipreps were performed using kits from TransGen Biotech Co. Ltd.

### MoClo DNA Assembly Reactions

Golden Gate Assembly reaction mixtures contained the following: ∼ 20 fmol of each DNA insert or plasmid, 1 μL of T4 DNA ligase buffer, 0.5 μL of T4 DNA ligase, 0.5 μL of BsaI-HF-v2 at 20,000 units/mL, or BsmBI-v2 at 10,000 units/mL, and nuclease-free water to bring the total volume up to 10 μL. Assembly reactions were performed in a thermocycler. Level 0 assemblies with only one part involved 1 h of digestion at 42 °C (BsmBI), 1 h of ligation at 16 °C, 20 min of a final digestion step, and 20 min of heat inactivation at 80 °C. Level 0 assemblies with more than one part and level 1 assemblies involved 25–35 cycles of digestion for 2 min (at 37 °C for BsaI or 42 °C for BsmBI) and ligation for 5 min, followed by the final digestion and heat inactivation steps. A “Part 234 GFP dropout vector” pYSD099 containing ConL1, ConR2, LEU2, CEN6/ARS4, and AmpR-ColE1 parts was prepared to simplify level 1 assemblies and constituted the backbone of most expression vectors. pYSD300, for coexpression of Aga1p, is an exception to this and has a HIS3 selectable marker gene. 2–5 μL of the assembly reaction was used for *E. coli* DH5α transformation. DNA from subsequent colonies was isolated, and restriction digests were performed for verification.

### Yeast Transformation

Yeasts were grown at 30 °C, shaking at 200 rpm overnight in YPD media. One mL of pelleted cells was added to 10 μL of 10 mg/mL single-stranded DNA that had been preincubated with 0.2–1 μg of plasmid. 500 μL of LiAc-TE-PEG (100 mM lithium acetate, 10 mM Tris-HCL pH 7.5, 1 mM EDTA, 40% w/v PEG 4000) was added to the cell pellet/DNA and incubated with occasional mixing for 15 min at room temperature. 50 μL of DMSO was added to the tube, followed by immediate heat shock at 42 °C for 15 min with occasional mixing. Cells were pelleted, resuspended in 50 μL of sterile water, and plated onto selective agar plates.

### Protein Expression, Western Blot Analysis, and Fluorescence Quantification

For protein expression experiments, 0.35 mL cultures of transformed cells were grown in selective synthetic media for 24 h and then diluted to 3.5 mL in buffered YPD media (2% bacto-tryptone, 1% yeast extract, and 2% dextrose (Formedium) supplemented with 0.85% MOPS free acid and 0.1 M dipotassium phosphate, adjusted to pH 7). They were grown in 13 mL tubes with vented caps (Sarstedt) at 30 °C, shaking at 200 rpm until saturated. Two mL of the saturated culture was centrifuged at 8000*g* for 5 min to pellet the cells, and a 0.22 μm filter was used to ensure the secreted protein containing supernatant was cell free. 20 μL of the cell-free supernatant was mixed with 6×-SDS-PAGE sample buffer. Cell pellets were lysed using the NaOH method. In brief, pellets were resuspended in 100 μL of Milli-Q water and 100 μL of 0.2 M NaOH added and incubated for 5 min. Samples were centrifuged at 8000*g* for 1 min, and pellets were resuspended in 50 μL of 2×-SDS-PAGE sample buffer. All samples were boiled at 95 °C for 5 min. Cell pellet samples were further diluted 1:20 in 2×-SDS-PAGE sample buffer. 10 μL of cell pellet samples and 20 μL of cell-free supernatant samples were used for SDS-PAGE analysis. Western blotting was performed following transfer onto Immobilon-FL PVDF membranes (Thermo Fisher Scientific) using an anti-histidine-tag primary antibody (Genscript A00186; 1:3000 dilution) and an IR800-conjugated secondary antibody (Li-Cor Biosciences). Images were acquired on an Odyssey imaging system and analyzed using Image Studio and Empiria Studio software (Li-Cor Biosciences). Western blots shown in the figures are representative of at least two independent experiments. To quantify mRuby2 fluorescence, 100 μL of supernatants or whole cultures was analyzed using a Tecan plate-reader with the excitation wavelength set at 559 nm and the emission wavelength set at 600 nm. In these cases, selective media were exclusively used for protein expression. Fluorescence was normalized for cell density by dividing fluorescence values by the OD_600_ values of whole cultures.

### Anti-GFP Nanobody Functionality Test

A 10 cm dish of HEK293T cells that had been transfected with the GFP-expressing plasmid pEGFP-N1 (Clontech) was washed with PBS and lysed by sonication on ice in PBS supplemented with 0.2% Triton X-100, 1 mM phenylmethylsulfonyl fluoride, 0.1 mg/mL lysozyme, and 0.1 mg/mL DNaseI and then centrifuged at 16,100*g* for 30 min at 4 °C to obtain the soluble cell lysate. Ni-NTA agarose beads (Neo Biotech) were prepared by washing 3 times in 1 mL of PBS with centrifugation at 1000*g*, blocked in 1% bovine serum albumin for 30 min at room temperature, and washed once with 1 mL of equilibration buffer (20 mM Tris pH 8, 500 mM NaCl, and 5 mM imidazole). A 50 mL saturated culture expressing the anti-GFP nanobody with the ECM14 SP was centrifuged at 4000*g* for 10 min to obtain the nanobody containing supernatant. Twenty-five μL of beads were incubated for 1 h at 4 °C with this supernatant and then washed with 3 × 1 mL PBS. The EGFP lysate was split between these beads and 25 μL of control beads that had not been incubated with the nanobody. The beads were incubated for 1 h at 4 °C and 5 × 1 mL washes with PBS were performed. 25 μL of 200 mM imidazole was incubated with the beads for 5 min to elute the proteins, which were mixed with 25 μL of 2× SDS-PAGE sample buffer.

### Flow Cytometry

For flow cytometry applications, 2 mL of yeast cultures was grown in 13 mL tubes, in appropriate selective synthetic media at 30 °C overnight, shaking at 200 rpm. Yeast cell density was measured. Approximately 1 × 10^6^ cells (assuming 1 OD_600_ ≈ 1.5 × 10^7^ cells/mL) were pipetted into each microcentrifuge tube and centrifuged for 3 min at 3500*g*. The supernatant was removed, and cells were resuspended in 100 μL of flow cytometry buffer (20 mM HEPES pH 7.5, 150 mM NaCl, 0.1% (w/v) bovine serum albumin, 5 mM maltose). Yeast were then incubated with 0.5 μg of Alexa Fluor 488-labeled HA-tag antibody (Biolegend) for 15 min at room temperature. An unstained sample was used as a control. After incubation, cells were pelleted again, resuspended in 500 μL of buffer, and analyzed by flow cytometry with an Accuri C6 instrument (BD Biosciences) using a 533/30 filter and an FL1 detector for green fluorescence.

### Displayed Anti-GFP Nanobody Functionality Test

Twenty-five milliliters of *E. coli* DH5α cells that had been transformed with the GFP Dropout Vector was grown at 37 °C with shaking at 200 rpm in LB liquid media with 50 μg/mL carbenicillin. The culture was centrifuged at 3500*g* for 5 min at 4 °C. The pellet was resuspended in PBS supplemented with 0.2% Triton X-100, 0.1 mg/mL lysozyme, and 0.1 mg/mL DNase and lysed by sonication on ice. The lysed cells were then centrifuged at 16,100*g* for 30 min at 4 °C to obtain the soluble cell lysate. The supernatant was filtered using a 0.22 μm pore size filter. For flow cytometry testing, yeast samples were prepared as described above and incubated for 15 min at room temperature with 100 μL of GFP-containing supernatant. After incubation, cells were pelleted, resuspended in 500 μL of flow cytometry buffer, and analyzed by flow cytometry as above.

### Plasmid Availability

Full details of all vectors used in this study are provided in Supporting Information File S1. Plasmids encoding the SP, TFP, and anchor protein parts, as well as a selection of assembled constructs that can serve as controls for protein secretion or surface display, have been depositted at Addgene and are being made available as a yeast secretion/display (YSD) plasmid kit (https://www.addgene.org/Paul_Young/). Fully annotated sequence files (in Genbank format) for the plasmids in the YSD toolkit have been added as Supporting Information to this article (File S2) and are also available through the Addgene webpage for each plasmid (navigate to Resource Information → Supplemental Documents).

## Supporting Information Available

Supporting Figures and Tables that include additional data and information described in the text: (Figure S1) Analysis of the TGCT overhang when used as part of YTK toolkit using the NEBridge GetSet tool from New England Biolabs; (Figure S2) evaluation of a panel of 13 secretion promoting sequences for the expression of yeast invertase; (Table S1) predicted and experimentally determined glycosylation sites in proteins of interest, FLAG-His tag, MFa pre-pro-SP, and TFP sequences. (PDF)
A supporting file that lists: oligonucleotides and gene fragments used; sequences of proteins of interest; descriptions of part plasmids used and generated; descriptions of assembled expression plasmids; and a list of pYSD plasmids made available through Addgene.org. (XLSX)
A supporting file with annotated sequences in GenBank format for the 34 plasmids included in the toolkit made available through Addgene.org. (ZIP)

## Abbreviations

AA: amino acid
ER: endoplasmic reticulum
FSC: forward light scattering
GFP: green fluorescent protein
GRAS: generally recognized as safe
MF: mating factor
MoClo: modular cloning
PBS: phosphate-buffered saline
POI: protein of interest
SEM: standard error of the mean
SP: signal peptide
SRP: signal recognition particle
TFP: translational fusion partners
YSD: yeast secretion/display
YTK: yeast toolkit

## References

[ref1] Nielsen J. Production of biopharmaceutical proteins by yeast. Bioengineered 2013, 4, 207–211. 10.4161/bioe.22856. 23147168 PMC3728191

[ref2] Gomes A. M. V.; Carmo T. S.; Carvalho L. S.; Bahia F. M.; Parachin N. S. Comparison of Yeasts as Hosts for Recombinant Protein Production. Microorganisms 2018, 6, 38 10.3390/microorganisms6020038. 29710826 PMC6027275

[ref3] Teymennet-Ramirez K. V.; Martinez-Morales F.; Trejo-Hernandez M. R. Yeast Surface Display System: Strategies for Improvement and Biotechnological Applications. Front. Bioeng. Biotechnol. 2021, 9, 794742 10.3389/fbioe.2021.794742. 35083204 PMC8784408

[ref4] Zahrl R. J.; Prielhofer R.; Ata O.; Baumann K.; Mattanovich D.; Gasser B. Pushing and pulling proteins into the yeast secretory pathway enhances recombinant protein secretion. Metab. Eng. 2022, 74, 36–48. 10.1016/j.ymben.2022.08.010. 36057427

[ref5] Owji H.; Nezafat N.; Negahdaripour M.; Hajiebrahimi A.; Ghasemi Y. A comprehensive review of signal peptides: Structure, roles, and applications. Eur. J. Cell Biol. 2018, 97, 422–441. 10.1016/j.ejcb.2018.06.003. 29958716

[ref6] Aza P.; Molpeceres G.; de Salas F.; Camarero S. Design of an improved universal signal peptide based on the alpha-factor mating secretion signal for enzyme production in yeast. Cell. Mol. Life Sci. 2021, 78, 3691–3707. 10.1007/s00018-021-03793-y. 33687500 PMC8038962

[ref7] Mori A.; Hara S.; Sugahara T.; Kojima T.; Iwasaki Y.; Kawarasaki Y.; Sahara T.; Ohgiya S.; Nakano H. Signal peptide optimization tool for the secretion of recombinant protein from Saccharomyces cerevisiae. J. Biosci. Bioeng. 2015, 120, 518–525. 10.1016/j.jbiosc.2015.03.003. 25912446

[ref8] Holec P. V.; Camacho K. V.; Breuckman K. C.; Mou J.; Birnbaum M. E. Proteome-Scale Screening to Identify High-Expression Signal Peptides with Minimal N-Terminus Biases via Yeast Display. ACS Synth. Biol. 2022, 11, 2405–2416. 10.1021/acssynbio.2c00101. 35687717 PMC9473667

[ref9] Besada-Lombana P. B.; Da Silva N. A. Engineering the early secretory pathway for increased protein secretion in Saccharomyces cerevisiae. Metab. Eng. 2019, 55, 142–151. 10.1016/j.ymben.2019.06.010. 31220665

[ref10] Lin-Cereghino G. P.; Stark C. M.; Kim D.; Chang J.; Shaheen N.; Poerwanto H.; Agari K.; Moua P.; Low L. K.; Tran N.; Huang A. D.; Nattestad M.; Oshiro K. T.; Chang J. W.; Chavan A.; Tsai J. W.; Lin-Cereghino J. The effect of alpha-mating factor secretion signal mutations on recombinant protein expression in Pichia pastoris. Gene 2013, 519, 311–317. 10.1016/j.gene.2013.01.062. 23454485 PMC3628533

[ref11] Rakestraw J. A.; Sazinsky S. L.; Piatesi A.; Antipov E.; Wittrup K. D. Directed evolution of a secretory leader for the improved expression of heterologous proteins and full-length antibodies in Saccharomyces cerevisiae. Biotechnol. Bioeng. 2009, 103, 1192–1201. 10.1002/bit.22338. 19459139 PMC2847895

[ref12] Fitzgerald I.; Glick B. S. Secretion of a foreign protein from budding yeasts is enhanced by cotranslational translocation and by suppression of vacuolar targeting. Microb. Cell Fact. 2014, 13, 125 10.1186/s12934-014-0125-0. 25164324 PMC4176846

[ref13] Bae J. H.; Sung B. H.; Kim H. J.; Park S. H.; Lim K. M.; Kim M. J.; Lee C. R.; Sohn J. H. An Efficient Genome-Wide Fusion Partner Screening System for Secretion of Recombinant Proteins in Yeast. Sci. Rep. 2015, 5, 12229 10.1038/srep12229. 26195161 PMC4508530

[ref14] Weber E.; Engler C.; Gruetzner R.; Werner S.; Marillonnet S. A modular cloning system for standardized assembly of multigene constructs. PLoS One 2011, 6, e16765 10.1371/journal.pone.0016765. 21364738 PMC3041749

[ref15] Bird J. E.; Marles-Wright J.; Giachino A. A User’s Guide to Golden Gate Cloning Methods and Standards. ACS Synth. Biol. 2022, 11, 3551–3563. 10.1021/acssynbio.2c00355. 36322003 PMC9680027

[ref16] Lee M. E.; DeLoache W. C.; Cervantes B.; Dueber J. E. A Highly Characterized Yeast Toolkit for Modular, Multipart Assembly. ACS Synth. Biol. 2015, 4, 975–986. 10.1021/sb500366v. 25871405

[ref17] Xue S.; Liu X.; Pan Y.; Xiao C.; Feng Y.; Zheng L.; Zhao M.; Huang M. Comprehensive Analysis of Signal Peptides in Saccharomyces cerevisiae Reveals Features for Efficient Secretion. Adv. Sci. 2023, 10, e2203433 10.1002/advs.202203433. PMC9839866 36478443

[ref18] Bae J. H.; Yun S. H.; Kim M. J.; Kim H. J.; Sung B. H.; Kim S. I.; Sohn J. H. Secretome-based screening of fusion partners and their application in recombinant protein secretion in Saccharomyces cerevisiae. Appl. Microbiol. Biotechnol. 2022, 106, 663–673. 10.1007/s00253-021-11750-9. 34971409

[ref19] Teufel F.; Almagro Armenteros J. J.; Johansen A. R.; Gislason M. H.; Pihl S. I.; Tsirigos K. D.; Winther O.; Brunak S.; von Heijne G.; Nielsen H. SignalP 6.0 predicts all five types of signal peptides using protein language models. Nat. Biotechnol. 2022, 40, 1023–1025. 10.1038/s41587-021-01156-3. 34980915 PMC9287161

[ref20] Kubala M. H.; Kovtun O.; Alexandrov K.; Collins B. M. Structural and thermodynamic analysis of the GFP:GFP-nanobody complex. Protein Sci. 2010, 19, 2389–2401. 10.1002/pro.519. 20945358 PMC3009406

[ref21] Lam A. J.; St-Pierre F.; Gong Y.; Marshall J. D.; Cranfill P. J.; Baird M. A.; McKeown M. R.; Wiedenmann J.; Davidson M. W.; Schnitzer M. J.; Tsien R. Y.; Lin M. Z. Improving FRET dynamic range with bright green and red fluorescent proteins. Nat. Methods 2012, 9, 1005–1012. 10.1038/nmeth.2171. 22961245 PMC3461113

[ref22] Huang D.; Shusta E. V. Secretion and surface display of green fluorescent protein using the yeast Saccharomyces cerevisiae. Biotechnol. Prog. 2008, 21, 349–357. 10.1021/bp0497482. 15801770

[ref23] Bilal M.; Ji L.; Xu S.; Zhang Y.; Iqbal H. M. N.; Cheng H. Bioprospecting and biotechnological insights into sweet-tasting proteins by microbial hosts-a review. Bioengineered 2022, 13, 9816–9829. 10.1080/21655979.2022.2061147. PMC9161876 35435127

[ref24] Ming D.; Hellekant G. Brazzein, a new high-potency thermostable sweet protein from Pentadiplandra brazzeana B. FEBS Lett. 1994, 355, 106–108. 10.1016/0014-5793(94)01184-2. 7957951

[ref25] Kim S. H.; Kang C. H.; Kim R.; Cho J. M.; Lee Y. B.; Lee T. K. Redesigning a sweet protein: increased stability and renaturability. Protein Eng. Des. Sel. 1989, 2, 571–575. 10.1093/protein/2.8.571. 2813335

[ref26] Spadaccini R.; Crescenzi O.; Tancredi T.; De Casamassimi N.; Saviano G.; Scognamiglio R.; Di Donato A.; Temussi P. A. Solution structure of a sweet protein: NMR study of MNEI, a single chain monellin. J. Mol. Biol. 2001, 305, 505–514. 10.1006/jmbi.2000.4304. 11152608

[ref27] Gupta R.; Brunak S. Prediction of glycosylation across the human proteome and the correlation to protein function. Pac. Symp. Biocomput. 2002, 310–322. 11928486

[ref28] Steentoft C.; Vakhrushev S. Y.; Joshi H. J.; Kong Y.; Vester-Christensen M. B.; Schjoldager K. T.; Lavrsen K.; Dabelsteen S.; Pedersen N. B.; Marcos-Silva L.; Gupta R.; Bennett E. P.; Mandel U.; Brunak S.; Wandall H. H.; Levery S. B.; Clausen H. Precision mapping of the human O-GalNAc glycoproteome through SimpleCell technology. EMBO J. 2013, 32, 1478–1488. 10.1038/emboj.2013.79. 23584533 PMC3655468

[ref29] González M.; Brito N.; Gonzalez C. High abundance of Serine/Threonine-rich regions predicted to be hyper-O-glycosylated in the secretory proteins coded by eight fungal genomes. BMC Microbiol. 2012, 12, 213 10.1186/1471-2180-12-213. 22994653 PMC3579731

[ref30] Neubert P.; Halim A.; Zauser M.; Essig A.; Joshi H. J.; Zatorska E.; Larsen I. S.; Loibl M.; Castells-Ballester J.; Aebi M.; Clausen H.; Strahl S. Mapping the O-Mannose Glycoproteome in Saccharomyces cerevisiae. Mol. Cell. Proteomics 2016, 15, 1323–1337. 10.1074/mcp.M115.057505. 26764011 PMC4824858

[ref31] Zielinska D. F.; Gnad F.; Schropp K.; Wisniewski J. R.; Mann M. Mapping N-glycosylation sites across seven evolutionarily distant species reveals a divergent substrate proteome despite a common core machinery. Mol. Cell 2012, 46, 542–548. 10.1016/j.molcel.2012.04.031. 22633491

[ref32] Reddy V. A.; Johnson R. S.; Biemann K.; Williams R. S.; Ziegler F. D.; Trimble R. B.; Maley F. Characterization of the glycosylation sites in yeast external invertase. I. N-linked oligosaccharide content of the individual sequons. J. Biol. Chem. 1988, 263, 6978–6985. 10.1016/S0021-9258(18)68592-8. 3284881

[ref33] Ziegler F. D.; Maley F.; Trimble R. B. Characterization of the glycosylation sites in yeast external invertase. II. Location of the endo-beta-N-acetylglucosaminidase H-resistant sequons. J. Biol. Chem. 1988, 263, 6986–6992. 10.1016/S0021-9258(18)68593-X. 3130375

[ref34] Saraiva A.; Carrascosa C.; Ramos F.; Raheem D.; Pedreiro S.; Vega A.; Raposo A. Brazzein and Monellin: Chemical Analysis, Food Industry Applications, Safety and Quality Control, Nutritional Profile and Health Impacts. Foods 2023, 12, 1943 10.3390/foods12101943. 37238762 PMC10217172

[ref35] Chen Z.; Li Z.; Yu N.; Yan L. Expression and secretion of a single-chain sweet protein, monellin, in Saccharomyces cerevisiae by an alpha-factor signal peptide. Biotechnol. Lett. 2011, 33, 721–725. 10.1007/s10529-010-0479-2. 21107648

[ref36] Liu J.; Yan D. Z.; Zhao S. J. Expression of monellin in a food-grade delivery system in Saccharomyces cerevisiae. J. Sci. Food Agric. 2015, 95, 2646–2651. 10.1002/jsfa.6997. 25382639

[ref37] Assadi-Porter F. M.; Aceti D. J.; Cheng H.; Markley J. L. Efficient production of recombinant brazzein, a small, heat-stable, sweet-tasting protein of plant origin. Arch. Biochem. Biophys. 2000, 376, 252–258. 10.1006/abbi.2000.1725. 10775410

[ref38] Neiers F.; Belloir C.; Poirier N.; Naumer C.; Krohn M.; Briand L. Comparison of Different Signal Peptides for the Efficient Secretion of the Sweet-Tasting Plant Protein Brazzein in Pichia pastoris. Life 2021, 11, 46 10.3390/life11010046. 33450886 PMC7828362

[ref39] Boyle P. M.; Burrill D. R.; Inniss M. C.; Agapakis C. M.; Deardon A.; DeWerd J. G.; Gedeon M. A.; Quinn J. Y.; Paull M. L.; Raman A. M.; Theilmann M. R.; Wang L.; Winn J. C.; Medvedik O.; Schellenberg K.; Haynes K. A.; Viel A.; Brenner T. J.; Church G. M.; Shah J. V.; Silver P. A. A BioBrick compatible strategy for genetic modification of plants. J. Biol. Eng. 2012, 6, 8 10.1186/1754-1611-6-8. 22716313 PMC3537565

[ref40] Neiers F.; Belloir C.; Poirier N.; Naumer C.; Krohn M.; Briand L. Comparison of Different Signal Peptides for the Efficient Secretion of the Sweet-Tasting Plant Protein Brazzein in Pichia pastoris. Life 2021, 11, 46 10.3390/life11010046. 33450886 PMC7828362

[ref41] Jo H. J.; Noh J. S.; Kong K. H. Efficient secretory expression of the sweet-tasting protein brazzein in the yeast Kluyveromyces lactis. Protein Expr. Purif. 2013, 90, 84–89. 10.1016/j.pep.2013.05.001. 23684772

[ref42] Park S. W.; Kang B. H.; Lee H. M.; Lee S. J.; Kim H. S.; Choi H. W.; Park T. J.; Kong K. H. Efficient brazzein production in yeast (Kluyveromyces lactis) using a chemically defined medium. Bioprocess Biosyst. Eng. 2021, 44, 913–925. 10.1007/s00449-020-02499-y. 33502625

[ref43] Obst U.; Lu T. K.; Sieber V. A Modular Toolkit for Generating Pichia pastoris Secretion Libraries. ACS Synth. Biol. 2017, 6, 1016–1025. 10.1021/acssynbio.6b00337. 28252957

[ref44] Rajkumar A. S.; Varela J. A.; Juergens H.; Daran J. G.; Morrissey J. P. Biological Parts for Kluyveromyces marxianus Synthetic Biology. Front. Bioeng. Biotechnol. 2019, 7, 97 10.3389/fbioe.2019.00097. 31134195 PMC6515861

[ref45] Loll-Krippleber R.; Sajtovich V. A.; Ferguson M. W.; Ho B.; Burns A. R.; Payliss B. J.; Bellissimo J.; Peters S.; Roy P. J.; Wyatt H. D. M.; Brown G. W. Development of a yeast whole-cell biocatalyst for MHET conversion into terephthalic acid and ethylene glycol. Microb. Cell Fact. 2022, 21, 280 10.1186/s12934-022-02007-9. 36587193 PMC9805092

[ref46] McMahon C.; Baier A. S.; Pascolutti R.; Wegrecki M.; Zheng S.; Ong J. X.; Erlandson S. C.; Hilger D.; Rasmussen S. G. F.; Ring A. M.; Manglik A.; Kruse A. C. Yeast surface display platform for rapid discovery of conformationally selective nanobodies. Nat. Struct. Mol. Biol. 2018, 25, 289–296. 10.1038/s41594-018-0028-6. 29434346 PMC5839991

[ref47] Cherf G. M.; Cochran J. R. Applications of Yeast Surface Display for Protein Engineering. Methods Mol. Biol. 2015, 1319, 155–175. 10.1007/978-1-4939-2748-7_8. 26060074 PMC4544684

[ref48] Chen Z.; Duan R.; Xiao Y.; Wei Y.; Zhang H.; Sun X.; Wang S.; Cheng Y.; Wang X.; Tong S.; Yao Y.; Zhu C.; Yang H.; Wang Y.; Wang Z. Biodegradation of highly crystallized poly(ethylene terephthalate) through cell surface codisplay of bacterial PETase and hydrophobin. Nat. Commun. 2022, 13, 7138 10.1038/s41467-022-34908-z. 36414665 PMC9681837

[ref49] Shaw W. M.; Khalil A. S.; Ellis T. A Multiplex MoClo Toolkit for Extensive and Flexible Engineering of Saccharomyces cerevisiae. ACS Synth. Biol. 2023, 12, 3393–3405. 10.1021/acssynbio.3c00423. 37930278 PMC10661031

[ref50] Otto M.; Skrekas C.; Gossing M.; Gustafsson J.; Siewers V.; David F. Expansion of the Yeast Modular Cloning Toolkit for CRISPR-Based Applications, Genomic Integrations and Combinatorial Libraries. ACS Synth. Biol. 2021, 10, 3461–3474. 10.1021/acssynbio.1c00408. 34860007 PMC8689691

[ref51] Simakin P.; Koch C.; Herrmann J. M. A modular cloning (MoClo) toolkit for reliable intracellular protein targeting in the yeast Saccharomyces cerevisiae. Microb. Cell 2023, 10, 78–87. 10.15698/mic2023.04.794. 37009624 PMC10054711

