# Supplementary material for: A Yeast Modular Cloning (MoClo) Toolkit Expansion for Optimization of Heterologous Protein Secretion and Surface Display in Saccharomyces cerevisiae
Source: ACS Synth Biol. 2024 Mar 14;13(4):1246–58. doi: 10.1021/acssynbio.3c00743 (PMC11036508; doi:10.1021/acssynbio.3c00743)
Supplement: Supplementary file 1 — sb3c00743_si_001.pdf [file sb3c00743_si_001.pdf]

SUPPORTING INFORMATION

(SUPPORTING FIGURES AND TABLES)

A yeast modular cloning (MoClo) toolkit expansion for optimization of heterologous protein secretion and surface display in *Saccharomyces cerevisiae*.

AUTHORS

Nicola M. O'Riordan[1], Vanja Jurić[1,2], Sarah K. O'Neill[1], Aoife P. Roche[1], Paul W. Young[1,2,*]

AFFILIATIONS

[1]School of Biochemistry and Cell Biology, University College Cork, Cork T12 YN60, Ireland
[2]AMBER Centre, Environmental Research Institute, University College Cork, Cork T23 XE10, Ireland
*Correspondence: p.young@ucc.ie

Figure S1

Overhang set

CCCT,AACG,TATG,ATCC,TGGC,GCTG,TACA,GAGT,CCGA,TGCT

Ligation frequency matrix

	CCCT	AGGG	AACG	CGTT	TATG	CATA	ATCC	GGAT	TGGC	GCCA	GCTG	CAGC	TACA	TGTA	GAGT	ACTC	CCGA	TCGG	TGCT	AGCA
CCCT	0	215	0	0	0	0	0	0	0	0	0	0	0	0	0	0	0	0	0	0
AGGG	215	0	0	0	0	0	0	0	0	0	0	0	0	0	0	0	0	0	0	0
AACG	0	0	0	271	0	0	0	0	0	0	0	0	0	0	0	0	0	0	0	0
CGTT	0	0	271	0	0	0	0	0	0	0	0	0	0	0	0	0	0	0	0	0
TATG	0	0	0	0	0	237	0	0	0	0	0	0	0	0	0	0	0	0	0	0
CATA	0	0	0	0	237	0	0	0	0	0	0	0	0	0	0	0	0	0	0	0
ATCC	0	0	0	0	0	0	0	284	0	0	0	0	0	0	0	0	0	0	0	0
GGAT	0	0	0	0	0	0	284	0	0	0	0	0	0	0	0	0	0	0	0	0
TGGC	0	0	0	0	0	0	0	0	0	243	0	0	0	0	0	0	0	0	0	0
GCCA	0	0	0	0	0	0	0	0	243	0	0	0	0	0	0	0	0	0	0	0
GCTG	0	0	0	0	0	0	0	0	0	0	0	173	0	0	0	0	0	0	0	0
CAGC	0	0	0	0	0	0	0	0	0	0	173	0	0	0	0	0	0	0	0	0
TACA	0	0	0	0	0	0	0	0	0	0	0	0	0	259	0	0	0	0	0	0
TGTA	0	0	0	0	0	0	0	0	0	0	0	0	259	0	0	0	0	0	0	0
GAGT	0	0	0	0	0	0	0	0	0	0	0	0	0	0	0	249	0	0	0	0
ACTC	0	0	0	0	0	0	0	0	0	0	0	0	0	0	249	0	0	0	0	0
CCGA	0	0	0	0	0	0	0	0	0	0	0	0	0	0	0	0	0	310	0	0
TCGG	0	0	0	0	0	0	0	0	0	0	0	0	0	0	0	0	310	2	0	0
TGCT	0	0	0	0	0	0	0	0	0	0	0	0	0	0	0	0	0	0	1	239
AGCA	0	0	0	0	0	0	0	0	0	0	0	0	0	0	0	0	0	0	239	0

Legend

- good Watson-Crick pair
- poor Watson-Crick pair
- high-count mismatch
- modest mismatch
- trace mismatch

Figure S1. Analysis of the TGCT overhang when used as part of YTK toolkit using the NEBridge GetSet tool from New England Biolabs The overhangs from the yeast modular cloning toolkit were analysed with TGCT replacing the standard Part 3a/3b overhang. Minimal mismatches are observed and Golden Gate Assembly is predicted to yield 99% of correctly-ligated products using this set of overhangs.

Figure S2

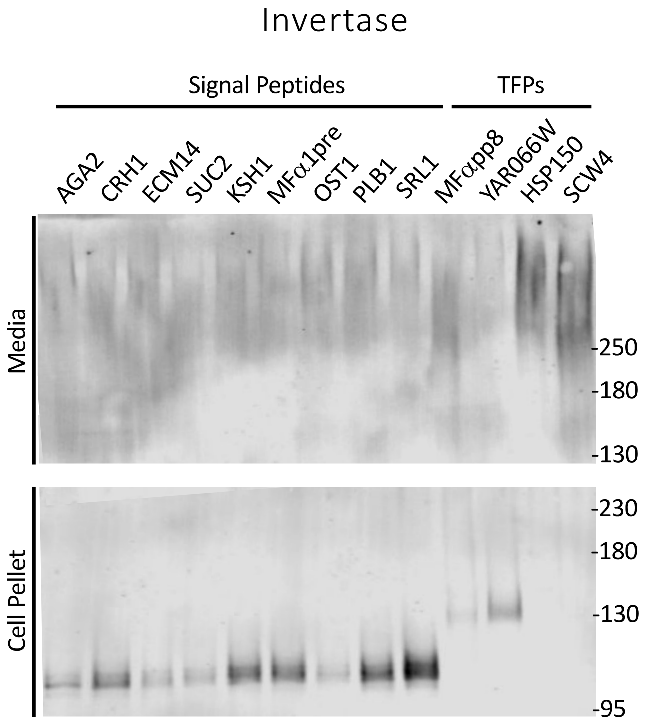

Figure S2. Evaluation of a panel of thirteen secretion promoting sequences for the expression of yeast invertase. Recombinant proteins were targeted for secretion using the indicated SPs and TFPs. Secreted and intracellular proteins were detected from the media and cell pellet respectively by western blotting for a carboxyl-terminal 6xHis tag. Secreted invertase was detected for all SP and TFP sequences as a very high molecular weight smear. This has been observed previously and was shown to be due to varying degrees of N-glycosylation on thirteen asparagine residues *(1, 2)*. Data presented is representative of two independent experiments.

Table S1

Table S1 Predicted and experimentally determined glycosylation sites in proteins of interest, FLAG-His tag, MFα pre-pro- SP and TFP sequences. N-linked glycosylation was predicted using *NetNGlyc1.0 (3)*. All Asn-Xaa-Ser/Thr sequons in the sequence are underlined. Asparagine residues predicted to be N-glycosylated using the default threshold of 0.5 are indicated in **bold red text.** O-linked glycosylation was predicted using *NetOGlyc4.0 (4)*. Serine and threonine residues predicted to be O-glycosylated are indicated in ***yellow bold italic text***. While the *NetOGlyc4.0* tool is intended to predict O-GalNAc (mucin type) glycosylation sites in mammalian proteins, an earlier version of this tool has been reported to have some ability to predict O-glycosylation sites in fungal proteins – particularly in highly O-glycosylated Ser/Thr rich regions *(5)*. Experimentally determined *S. cerevisiae* O-mannosylation sites *(6)* are indicated by cyan highlighting. Green highlighting indicates experimentally determined *S. cerevisiae* N-glycosylation sites*(1, 2, 6, 7)*. Note that experimental data is only available for endogenous *S. cerevisiae* proteins and that some regions of these proteins may not be well represented in mass spectrometry derived data. Pink highlighting indicates signal peptide pre- regions.

**SUC2 / Invertase:** 12 N-glycosylated & 3 O-glycosylated sites predicted; 13 N-glycosylated & 1 O-glycosylated sites experimentally validated<br>MLLQAFLFLLAGFAAKISASMTNETSDRPLVHFTPNKGWMNDPNGLWYDEKDAKWHLYFQYNPNDTVWGTPLFWGHATSD 80<br>DLTNWEDQPIAIAPKRNDSGAFSGSMVVDYNNTSGFFNDTIDPRQRCVAIWTYNTPESEEQYISYSLDGGYTFTEYQKNP 160<br>VLAANSTQFRDPKVFWYEPSQKWIMTAAKSQDYKIEIYSSDDLKSWKLESAFANEGFLGYQYECPGLIEVPTEQDPSKSY 240<br>WVMFISINPGAPAGGSFNQYFVGSFNGTHFEAFDNQSRVVDFGKDYYALQTFFNTDPTYGSALGIAWASNWEYSAFVPTN 320<br>PWRSSMSLVRKFSLNTEYQANPETELINLKAEPILNISNAGPWSRFATNTTLTKANSYNVDLSNSTGTLEFELVYAVNTT 400<br>QTISKSVFADLSLWFKGLEDPEEYLRMGFEVSASSFFLDRGNSKVKFVKENPYFTNRMSVNNQPFKSENDLSYYKVYGLL 480<br>DQNILELYFNDGDVVSTNTYFMTTGNALGSVNMTTGVDNLFYIDKFQVREVK
**Anti-GFP Nanobody:** No N-glycosylated or O-glycosylated sites predicted<br>QVQLVESGGALVQPGGSLRLSCAASGFPVNRYSMRWYRQAPGKEREWVAGMSSAGDRSSYEDSVKGRFTISRDDARNTVY 80<br>LQMNSLKPEDTAVYYCNVNVGFEYWGQGTQVTVSSGSYPYDVPDYA
**mRuby2:** No N-glycosylated & 2 O-glycosylated sites predicted<br>MVSKGEELIKENMRMKVVMEGSVNGHQFKCTGEGEGNPYMGTQTMRIKVIEGGPLPFAFDILATSFMYGSRTFIKYPKGI 80<br>PDFFKQSFPEGFTWERVTRYEDGGVVTVMQDTSLEDGCLVYHVQVRGVNFPSNGPVMQKKTKGWEPNTEMMYPADGGLRG 160<br>YTHMALKVDGGGHLSCSFVTTYRSKKTVGNIKMPGIHAVDHRLERLEESDNEMFVVQREHAVAKFAGLGGGMDELYK 240
**Brazzein:** No N-glycosylated or O-glycosylated sites predicted<br>DKCKKVYENYPVSKCQLANQCNYDCKLDKHARSGECFYDEKRNLQCICDYCEY
**scMonellin:** No N-glycosylated or O-glycosylated sites predicted<br>GEWEIIDIGPFTQNLGKFAVDEENKIGQYGRLTFNKVIRPCMKKTIYEENGFREIKGYEYQLYVYASDKLFRADISEDYK 80<br>TRGRKLLRFNGPVPPP
**3xFLAG-6xHis:** No N-glycosylated or O-glycosylated sites predicted<br>GSDYKDDDDKDYKDDDDKDYKDDDDKGHHHHHH
**Mating factor-α pre-pro-(MFαppWT):** 3 N-glycosylated & 1 O-glycosylated sites predicted (present in all three MFα variants studied)<br>MRFPSIFTAVLFAASSALAAPVNTTTEDETAQIPAEAVIGYLDLEGDFDVAVLPFSNSTNNGLLFINTTIASIAAKEEGV 80<br>SLDKREA
**YAR066W TFP:**1 N-glycosylated & 15 O-glycosylated sites predicted; 5 O-glycosylated sites determined experimentally<br>MFNRFNKFQAAVALALLSRGALGDSYTNSTSSADLSSITSVSSASASATASDSLSSSDGTVYLPSTTISGDLTVTGKVIA 80<br>TEAVEVAAGGKLTLLDGEKYVFSSD
**HSP150 TFP:** No N-glycosylated & 45 O-glycosylated sites predicted (No Asparagines); 28 O-glycosylated sites determined experimentally<br>MQYKKTLVASALAATTLAAYAPSEPWSTLTPTATYSGGVTDYASTFGIAVQPISTTSSASSAATTASSKAKRAASQIGDG 80<br>QVQAATTTASVSTKSTAAAVSQIGDGQIQATTKTTAAAVSQIGDGQIQATTKTTSAKTTAAAVSQISDGQIQATTTTLAP 160<br>K
**SCW4 TFP:** No N-glycosylated & 20 O-glycosylated sites predicted; 19 O-glycosylated sites determined experimentally<br>MRLSNLIASASLLSAATLAAPANHEHKDKRAVVTTTVQKQTTIIVNGAASTPVAALEENAVVNSAPAAATSTTSSAASVA 80<br>TAAASSSENNSQVSAAASPASSSAATSTQSS

References:

(1) Reddy, V. A., Johnson, R. S., Biemann, K., Williams, R. S., Ziegler, F. D., Trimble, R. B., and Maley, F. (**1988**) Characterization of the glycosylation sites in yeast external invertase. I. N-linked oligosaccharide content of the individual sequons, *J Biol Chem 263*, 6978-6985.

(2) Ziegler, F. D., Maley, F., and Trimble, R. B. (**1988**) Characterization of the glycosylation sites in yeast external invertase. II. Location of the endo-beta-N-acetylglucosaminidase H-resistant sequons, *J Biol Chem 263*, 6986-6992.

(3) Gupta, R., and Brunak, S. (**2002**) Prediction of glycosylation across the human proteome and the correlation to protein function, *Pac Symp Biocomput*, 310-322.

(4) Steentoft, C., Vakhrushev, S. Y., Joshi, H. J., Kong, Y., Vester-Christensen, M. B., Schjoldager, K. T., Lavrsen, K., Dabelsteen, S., Pedersen, N. B., Marcos-Silva, L., Gupta, R., Bennett, E. P., Mandel, U., Brunak, S., Wandall, H. H., Levery, S. B., and Clausen, H. (**2013**) Precision mapping of the human O-GalNAc glycoproteome through SimpleCell technology, *EMBO J 32*, 1478-1488.

(5) Gonzalez, M., Brito, N., and Gonzalez, C. (**2012**) High abundance of Serine/Threonine-rich regions predicted to be hyper-O-glycosylated in the secretory proteins coded by eight fungal genomes, *BMC Microbiol 12*, 213.

(6) Neubert, P., Halim, A., Zauser, M., Essig, A., Joshi, H. J., Zatorska, E., Larsen, I. S., Loibl, M., Castells-Ballester, J., Aebi, M., Clausen, H., and Strahl, S. (**2016**) Mapping the O-Mannose Glycoproteome in Saccharomyces cerevisiae, *Mol Cell Proteomics 15*, 1323-1337.

(7) Zielinska, D. F., Gnad, F., Schropp, K., Wisniewski, J. R., and Mann, M. (**2012**) Mapping N-glycosylation sites across seven evolutionarily distant species reveals a divergent substrate proteome despite a common core machinery, *Mol Cell 46*, 542-548.
